# Outbreak of delta variant SARS-CoV-2 virus on a psychogeriatric ward in Helsinki, Finland, August 2021: two-dose vaccination reduces mortality and disease severity amongst the elderly

**DOI:** 10.1017/S0950268822000589

**Published:** 2022-04-20

**Authors:** Adnan Malik, Laura Lehtola, Sanna Isosomppi, Teemu Smura, Jaana Saarenheimo, Veli-Jukka Anttila, Eeva Särelä

**Affiliations:** 1 Suursuo Hospital, Social Services and Healthcare Division, Helsinki, Finland; 2 Infectious Diseases Unit, Helsinki University Hospitals, Helsinki, Finland; 3 Epidemiological Operations Unit, Social Services and Healthcare Division, Helsinki, Finland; 4 Department of Virology, Faculty of Medicine, University of Helsinki, Helsinki, Finland

**Keywords:** corona, Covid 19, vaccination, geriatrics, inpatient

## Abstract

We describe an outbreak of delta variant SARS-CoV-2 on a psychogeriatric ward of elderly patients. Retrospectively collected data was analysed using Fisher's exact test to assess the association between patients’ vaccination status and infection rates, severity of disease and mortality. Vaccination with two doses was shown to reduce severity of disease (5% *vs.* 75%, *p* < 0.001) and mortality (5% *vs.* 50%, *p* < 0.018) amongst an elderly inpatient population during an outbreak of delta variant SARS-CoV-2. Vaccination should be encouraged in elderly care institutions. Furthermore, adequate vaccination in elderly care institutions is an important consideration in current booster (third/fourth) dose schedules.

## Introduction

Prior to the emergence of the omicron variant, the delta variant was the most transmissible variant of SARS-CoV-2 (severe acute respiratory syndrome coronavirus 2). Development of SARS-CoV-2 vaccinations began before the emergence of the delta variant. Age is a major risk factor for contracting SARS-CoV-2 virus, developing severe disease and mortality [1]. Two-dose vaccination has been shown to be effective against the delta variant [2] and third-dose booster doses have already started to be given. Reduced effectiveness of vaccination has been to be linked to advancing age [3–5]. We describe an outbreak of the delta variant on a psychogeriatric ward in which two-dose vaccination reduced mortality and severity of disease amongst elderly inpatients.

## Description of outbreak

**Fig. 1.**
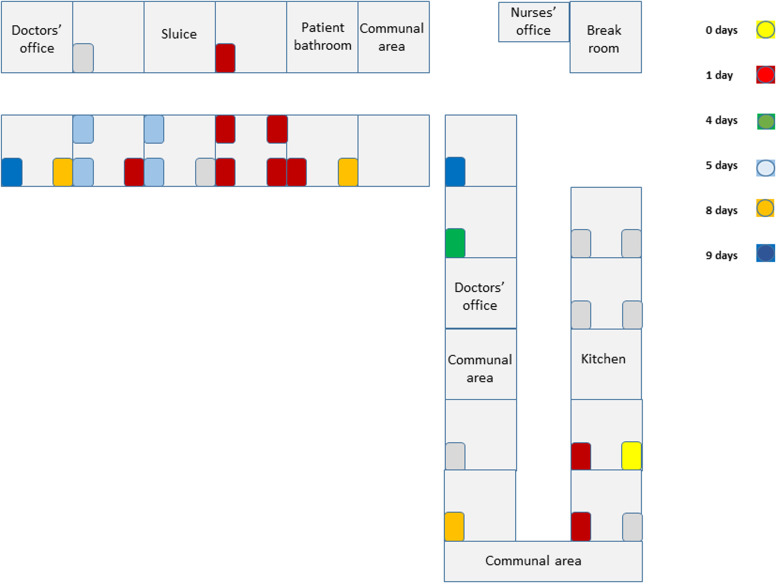
Topographic view of ward. Patient beds are coloured according to time (days since the index case) that the patient tested positive for SARS-CoV-2 virus.

The outbreak occurred on a psychogeriatric ward of a rehabilitation hospital in Helsinki, Finland. The hospital contains eight wards, with 200 patient beds. Patients are elderly, usually with underlying diseases requiring rehabilitation following acute care. Two of the eight wards are palliative care wards. The psychogeriatric ward consists of 28 patient beds on two different wings (Fig. 1). At the time of the outbreak, there were 27 inpatients. Patients have advanced mental care issues requiring close contact with healthcare personnel. Patients move freely on the ward and have use of communal spaces. Average lengths of stay are 1–3 months. In accordance with coronavirus regulations, surgical facemasks have been in use during patient contact and in communal areas since spring 2020. Where possible, healthcare personnel and patients obey the 2 m distancing rule.

On 18 August 2021, a frequently wandering 71-year-old patient developed a runny nose and 3 days later, following development of a fever, tested positive for SARS-CoV-2. All patients on the ward were subsequently tested. Nine patients tested positive during the first round of screening. At this point an epidemic on the ward was declared. Personnel protective equipment was upgraded to include droplet and contact precautions. During patient contact healthcare personnel used FFP2-respirator masks, eye protection (protective glasses/visors), long-sleeved protective aprons/jackets and surgical gloves. The only exception to this rule was if the healthcare worker was entering the patient's room for the sole purpose of delivering medications to the patient. In these instances, use of protective aprons/jackets was omitted. Patients were confined to their own rooms as best as possible. The ward closed to new admissions. All non-essential traffic on the ward (healthcare workers from other wards, visitors from outside the hospital) was restricted. The index patient had laboratory confirmation of infection with the delta variant on 22 August 2021. Six symptomatic SARS-CoV-2-positive patients were transferred to a cohort ward in another hospital. Four mildly symptomatic/asymptomatic SARS-CoV-2-positive patients were quarantined in their own rooms. Any patients with symptoms of infection were tested immediately, and all asymptomatic patients were screened a further four times. In total, 20 patients tested positive for SARS-CoV-2 during the outbreak. No new cases were identified in the final two screening rounds performed in September 2021 and the ward re-opened to new patients on 13 September 2021.

Eleven inadequately vaccinated healthcare personnel (five vaccinated with one dose, six unvaccinated) were quarantined at home. Twenty-four out of 29 healthcare personnel agreed to be screened, of which two tested positive. One was unvaccinated and the other had received only one vaccine dose.

## Methods

During the outbreak, patients were tested using nasopharyngeal SARS-CoV-2 RT-PCR (real-time reverse transcriptase). Genetic sequencing was performed on the index case to confirm infection with the delta variant (B.1.617.2). Further sequencing was requested for all positive samples to identify specific gene mutations and their epidemiological context.

We collected data on patients and healthcare personnel on the ward after the outbreak. After determining vaccination status, infection rates and outcomes, we analysed the data using Fisher's exact test to determine whether there was a statistically significant correlation between vaccination status and contracting infection, death rates and disease severity.

Two-dose vaccinated patients were defined as those having received two doses of a nationally recommended vaccine (AstraZeneca Vaxzevria, BioNTech-Pfizer Comirnaty, Moderna Spikevax), with at least one week having elapsed since the second dose. Patients having receiving less than two doses and patients less than a week from their second dose at time of infection were defined as inadequately vaccinated.

Severe disease was defined as presence of one or more of the following; breathing difficulties requiring supplemental oxygen or clinical/radiological evidence of pneumonia [6]. These criteria were adapted from the World Health Organization's criteria and applied to the study population of an elderly cohort in a rehabilitation hospital with ceilings of care restricted to Level 1 [7] care in most cases.

## Results

**Fig. 2.**
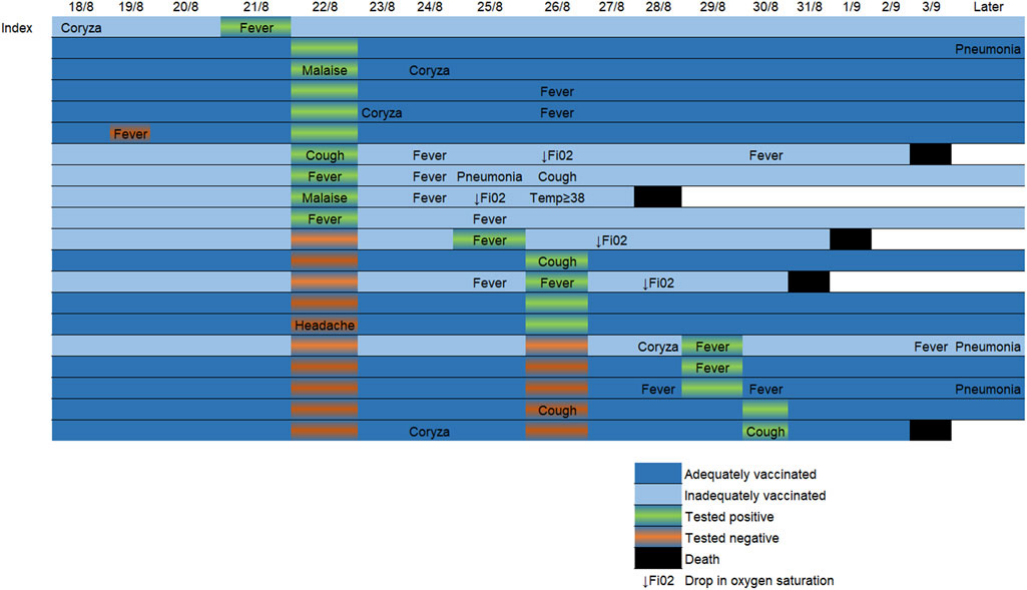
Timeline showing temporal relationship of possible SARS-CoV-2 symptoms, test dates/results and clinical course of COVID-19 positive patients during the epidemic. “Later” column depicts relevant information included in the follow-up period during dates not represented in the timeline.

Before the outbreak, 19/27 patients (70%) were two-dose vaccinated and eight (30%) were inadequately vaccinated. No patient had previously been diagnosed with SARS-CoV-2 infection. Age range of patients on the ward was 70–93, mean 82. As well as dementia and psychiatric risk factors, all the patients on the ward had at least one currently recognised additional risk factor for severe disease [8]. Patients' vaccination status is shown in Table 1. In total, 18/29 healthcare personnel were two-dose vaccinated, five had received one dose and six were unvaccinated. 20/27 patients tested positive for SARS-CoV-2. 12/19 two-dose vaccinated and 8/8 of those inadequately vaccinated tested positive. Disease was asymptomatic or mild in 10/12 of those vaccinated with two doses and in 2/8 of those inadequately vaccinated. Only one patient had documented evidence of a possible SARS-CoV-2 symptom prior to the index case's diagnosis. This occurred one day after the index case's likely initial symptoms and the non-index patient tested negative at the time. Two of the adequately vaccinated patients had evidence of severe disease compared to six of the inadequately vaccinated patients. One of the 19 two-dose vaccinated patients died, whereas four of eight inadequately vaccinated patients died. All four of the inadequately vaccinated mortality cases had at least one marker of severe disease. The adequately vaccinated mortality case had no evidence of severe disease prior to death. In all but one of the mortality cases, mortality occurred within one week of testing positive. The longest time from positive test to death was 13 days. All five mortalities occurred in less than 2 weeks of symptom onset. Figure 2 shows the temporal relationship between positive test, symptom onset and death in SARS-CoV-2-positive patients.

**Table 1.** Vaccination, infection rates and clinical course during the outbreak

	Two-dose vaccinated		Inadequately vaccinated	
	*n*	% (of total)	*n*	% (of total)
Total	19		8	
SARS-CoV-2 PCR-positive	12	63%	8	100%
Symptomless infection	2	11%	0	0%
Non-severe disease	9	47%	2	25%
Died	1	5%	4	50%

Two-dose vaccinated = patients who had received two doses of a nationally recommended vaccine. Inadequately vaccinated = patients having received no doses, one dose or those less than a week from their second dose.

Mortality amongst the two-dose vaccinated patients was statistically significantly lower than in those inadequately vaccinated (5% *vs.* 50%, *p* < 0.018). Severe disease was also statistically significantly lower in those vaccinated with two doses (11% *vs.* 75%; *p* < 0.01).

There was no statistical significant difference between infection rates in the two-dose vaccinated (63%) and inadequately vaccinated (100%) patients (*p* = 0.068) observed in this study.

**Fig. 3.**
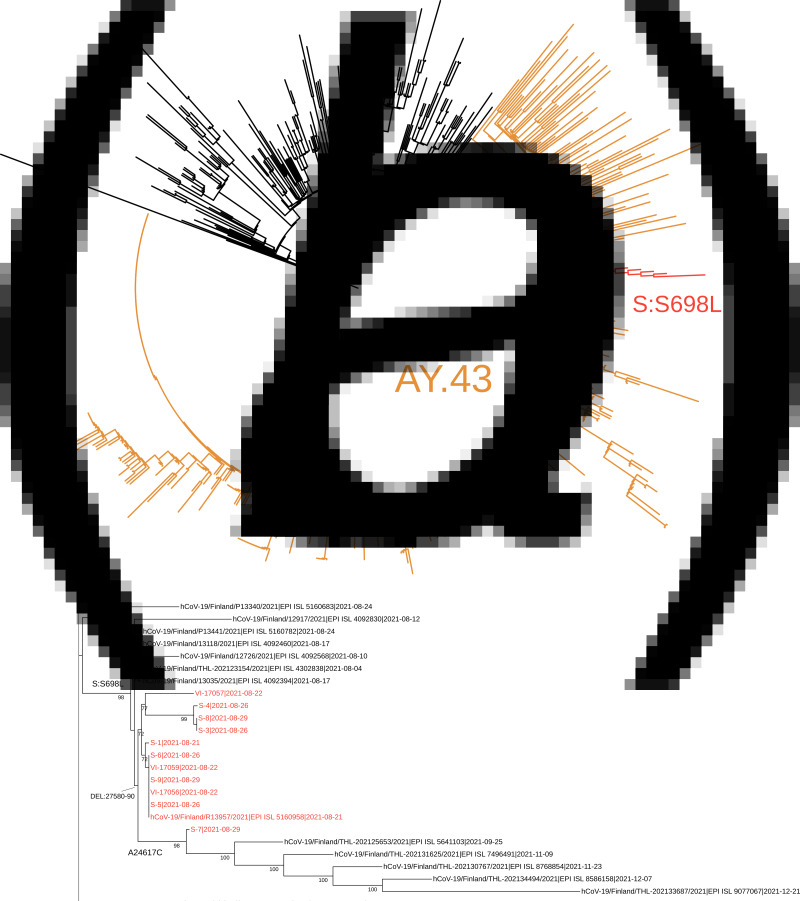
The complete tree is shown in figure 3a and the subtree containing the sequences from this outbreak is shown in figure 3b. In figure 3a the representatives of AY.43 lineage are shown in orange and the subcluster with spike protein substitution S698L is shown in red. In figure 3b, the sequences from this outbreak are marked with red and the signature mutations/deletion are shown in respective branches. The tree nodes with bootstrap support less than 70 were collapsed in this subtree.

Of the 20 samples analysed further gene sequencing was possible in 11 of the samples. Nine samples were not adequate for further gene sequencing. Of those analysed, presence of the lineage AY.43 delta variant was confirmed in all. The phylogenetic analysis suggested that these sequences are members of a subcluster that contains S698L substitution in spike protein. This substitution is present also in two samples from random population surveillance collected prior to this outbreak. An ORF7B deletion 27580-27590 was present in all analysed samples and was specific to this outbreak. It had not previously been observed in the Finnish population. A synonymous A24617C mutation was detected in one sequence. This mutation was found in five population surveillance samples collected after this outbreak (25 September to 21 December), suggesting further spread in the community. In addition, two singleton mutations (G15982T and spike protein D228N) as well as one mutation C4587T common to three sequences were detected. Phylogenetic trees are shown in Figures 3a and 3b.

## Discussion

The SARS-CoV-2 delta variant has been shown to spread easily amongst inadequately vaccinated individuals despite the appropriate use of surgical facemasks [9]. Vaccination with two doses provides protection against delta variant SARS-CoV-2 (88%), whereas protection following one dose is reported to be around 36% [10]. Those especially at risk following one dose include the elderly [11].

Although the rate of infection (63%) amongst individuals vaccinated with two doses observed in this outbreak was higher than would be expected (~10% [12, 13]), vaccination with two doses was shown to significantly reduce mortality and disease severity. This is despite the lack of statistically significant difference in infection rates between adequately and inadequately vaccinated patients seen in this study. Possible reasons for a high rate of infection include difficulties in isolating patients on a psychogeriatric ward and a delay in diagnosis. The index case was a mobile (despite restrictions), confused patient who was tested for SARS-CoV-2 when fever developed. However, the index patient's first possible SARS-CoV-2 symptom of runny-nose was 3 days prior to testing. This delay in diagnosis increased the ward's other patients' exposure to the virus as the index patient continued to move freely on the ward whilst being unknowingly infectious. Previous studies have shown three-quarters of transmission occurring 2 days before symptom onset to 2 days after [14, 15]. The ward was closed very soon (within one day) after confirmation of the index case, isolating the index case to the wing of the ward in which he had already exposed others to the SARS-CoV-2 virus. The index patient had been on the ward for one month prior to testing positive for SARS-CoV-2 and had been treated in two other hospitals prior to transfer onto the psychogeriatric ward. This suggests that the index patient was infected whilst on the hospital ward, most likely by an asymptomatic visitor/healthcare worker. Gene sequencing revealed the presence of an ORF7B deletion throughout the study population, which supports the idea that all cases originated from the same source and not multiple sources. A third reason for a higher rate of infection amongst the study population was the low level of vaccination with two doses amongst patients (70%) and healthcare personnel (56%). Whilst there was no statistically significant difference in infection rates between the adequately vaccinated and inadequately vaccinated patients in this cohort, the overall lower-than-desired vaccination coverage in this population would lead to a higher rate of infection compared to other observed populations. A proportion of the inadequately vaccinated patients had transferred between care facilities in the preceding months, leading to breaks in continuity of care and failure to administer vaccinations on time. Some patients refused to be vaccinated. There was also a break in the weekly vaccination schedule due to summer holidays. Also, the outbreak coincided with the introduction of trainee healthcare personnel to the ward, many of whom did not meet the vaccination programme criteria to be vaccinated with two doses at the time of the outbreak. A proportion of healthcare personnel declined vaccination. Asymptomatic carriage amongst those unvaccinated would lead to a higher prevalence of the virus overall on the ward. This would in turn increase patients' exposure to the virus, and subsequent chance of developing infection. None of the current vaccines have 100% efficacy [16].

Prior to vaccination, mortality from SARS-CoV-2 was close to 50% in elderly care facilities [17]. In our study, overall mortality was much lower amongst SARS-CoV-2-positive patients (1/19, 5%) that had been vaccinated with two doses, with mortality amongst inadequately vaccinated patients being comparable to the pre-vaccine era (50%). These results were observed in a population with universal prevalence of risk factors for severe SAR-CoV-2 disease. This encourages the administration of two-dose vaccine to elderly patients to reduce mortality and severity of disease in outbreaks caused by the delta variant, particularly with emerging evidence of waning immunity after one dose [18]. Furthermore, this study highlights the need for adequate vaccination in elderly care facilities when considering vaccination schedules for booster (third/fourth) doses (which started in Finland October 2021).

## Data Availability

The data collected from this study is available from the corresponding author upon reasonable request.
